# Nanovectors Design for Theranostic Applications in Colorectal Cancer

**DOI:** 10.1155/2019/2740923

**Published:** 2019-10-01

**Authors:** Riccardo Rampado, Sara Crotti, Paolo Caliceti, Salvatore Pucciarelli, Marco Agostini

**Affiliations:** ^1^First Surgical Clinic Section, Department of Surgical, Oncological and Gastroenterological Sciences, University of Padua, Via Nicolò Giustiniani 2, 35128 Padua, Italy; ^2^Nano-Inspired Biomedicine Laboratory, Institute of Paediatric Research- Città della Speranza, Corso Stati Uniti 4, 35127 Padua, Italy; ^3^Department of Pharmaceutical and Pharmacological Sciences, University of Padua, Via Marzolo, 5, 35131 Padua, Italy

## Abstract

Colorectal cancer (CRC) is a diffused disease with limited therapeutic options, none of which are often curative. Based on the molecular markers and targets expressed by the affected tissues, numerous novel approaches have been developed to study and treat this disease. In particular, the field of nanotechnology offers an astonishingly wide array of innovative nanovectors with high versatility and adaptability for both diagnosis and therapy (the so called “theranostic platforms”). However, such complexity can make the selection of a specific nanocarrier model to study a perplexing endeavour for the biomedical scientist or clinician not familiar with this field of inquiry. This review offers a comprehensive overview of this wide body of knowledge, in order to outline the essential requirements for the clinical viability evaluation of a nanovector model in CRC. In particular, the differences among the foremost designs, their specific advantages, and technological caveats will be treated, never forgetting the ultimate endpoint for these systems development: the clinical practice.

## 1. Colorectal Cancer (CRC)

### 1.1. Epidemiology

CRC is one of the most common cancers in both males and females. The yearly deaths caused by CRC are counted by tens of thousands of people, accounting for 10% of cancer-related mortality. CRC risk has been related to the increase in average population age and sedentary lifestyle. Indeed, environmental risk factors for the CRC development include: being overweight or obese, lack of physical activity, high meat and alcohol consumption, and smoking.

In CRC, the overall 5-year survival rate is ∼65%, with longer time survival only modestly improved in the last decades [1]. The main symptoms associated with CRC are blood in the stool, abdominal discomfort and pain, weight loss, and asthenia.

### 1.2. Pathogenesis

Colorectal cancer aetiology, as it is the case for many other tumours, involves both genetic and environmental risk factors. Among the various CRC causes, ∼75% can be attributed to sporadic disease, with no apparent predisposing aetiology. Although genetic factors account for a minority of CRC cases, a positive history of CRC has a strong correlation with increased incidence [1].

Both genetic and epigenetic alterations are involved in CRC development. The dominant model of carcinogenic mechanism for CRC is the so-called adenoma-carcinoma sequence, a multiple stages mechanism that involves a specific sequence of event for tumor initiation and progression. CRC-related genetic alterations appear to rely mostly on three mechanisms: chromosomal instability, high microsatellite instability, or CpG island methylator phenotype.

CRCs with chromosomal instability seem to affect mostly the left colon and are characterized by several chromosomal alteration including trisomy or deletions of chromosome arms. These tumours represent the wide majority of CRCs, with 70% prevalence. The second mechanism relies on the silencing of the DNA mismatch repair system that leads to a quick accumulation of numerous mutations, including genes encoding for membrane-associated proteins. This mechanism produces highly antigenic tumor cells and is characterized by a greater immune cell infiltrate, allowing for a better patient prognosis. Finally, the methylation of specific genes is the third mechanism, which characterizes tumours mostly located at the proximal colon [2, 3].

If the traditional adenoma-carcinoma sequence accounts for ∼50–60% of CRCs, alternative developmental trajectories, such as the serrated pathway (serrated adenomatous lesions which frequently display BRAF mutations) and chronic inflammation (i.e., colitis-associated CRC development, with TP53 mutations), are thought to be responsible for the other CRCs [4].

Chronic inflammation has been associated to the development and maintenance of different neoplasms in every phase of their natural history. The chronic inflammation which sustains CRC initiation and maintenance can be caused by environmental factors [5] or by pre-existing diseases, including chronic uncontrolled inflammatory bowel disease. Inflammation can promote genetic instability, which is a substrate for the acquisition of oncogenic mutations [6]. Furthermore, inflammation induces the accumulation of immune cells such as “antitumor” M1 macrophages (producing ROSs, IL-1*β*, TNF*α*, IL-6, and IL-12) in the tumor microenvironment, which are then reprogrammed by the tumor cells to a M2 phenotype. In particular, M2 polarized tumor-associated macrophages (TAMs) can promote cell proliferation, inhibiting apoptosis and promoting angiogenesis and metastasis, either by direct contact or by the production of immune-modulatory cytokines (e.g., IL-10 and TGF-*β*) [7]. Furthermore, a constant state of inflammation causes a prolonged exposition of healthy cells to ROSs, which can further damage DNA [8]. Cytokines can lead to the dedifferentiation of tumor cells into cancer staminal cells which are of critical relevance in phenomena such as cancer promotion, drug resistance, and recurrence after therapy [9, 10].

Inflammation, often in conjunction with hypoxia, can stimulate angiogenesis, triggered by the production of VEGFA by TAMs [11, 12] and the extracellular matrix (ECM) remodelling by secreted matrix metalloproteases [13]. Finally, at the tumor microenvironment, the establishment of an immune-suppressive milieu by the induction of tryptophan metabolism through the kynurenine pathway plays an important role in tumor immune evasion [14].

### 1.3. Current Diagnostic and Therapeutic Approaches

CRC population screening and diagnosis are performed with many different techniques. In people who have no symptoms, fecal tests are employed for hidden blood in the stool. Immunochemical fecal occult blood test (iFOBT) and guaiac-based fecal occult blood test (gFOBT) are low invasive tests largely employed in screening programs. However, these tests are not able to discriminate if the blood is from the colon or from other parts of the gastrointestinal tract. A stool DNA test, which searches for specific DNA mutations, ensures more specificity [15]. Blood-based screening tests, such as the detection of CEA antigen, the most well-known tumor markers for CRC, are helpful for diagnosis. Recently, the FDA has approved the use of some novel techniques such as CellSearch™ which allows for the detection of circulating tumor cells; however, these techniques are costly and are not easy to implement routinely.

Usually, colonoscopy is reserved to symptomatic patients or, in fecal tests positive patients, is used only as second-line treatment for routine screening. However, this approach is invasive, has some problems of patient compliance and possesses some risks (including anaesthesia and possible tissue damage). If this approach is not possible, alternative techniques are available, such as barium enemas for X-ray rectal outlining, or more complete techniques including virtual colonoscopy and computed tomographic colonography, which, however, require colon cleaning before the analysis and can detect non-neoplastic abnormalities.

Treatments for CRC are based largely on the tumor stage. The most important approach to CRC therapy as first-line treatment is the surgical resection of the tumor mass, but it is applicable only if the tumor mass is well-defined, and in some cases, the physical damage to the tumor mass itself can spark some metastasis. Radiotherapy is used before surgery (often in conjunction with neoadjuvant therapy) in order to reduce the tumor mass and make the tumor margins more defined, or after, in order to better destroy any remaining tumor cells and avoid relapses.

Chemotherapy is a third, therapeutic approach that can give great benefits against CRC, especially in more advanced stages, but is characterized by severe side effects. Chemotherapy relies on three main drugs: 5-fluorouracil (5-FU) is an antimetabolite which is able to inhibit the production of thymidine by irreversibly blocking the rate limiting step of its synthesis in the thymidylate synthase pathway. 5-FU is also available orally as capecitabine (prodrug) and can often be coadministered with leucovorin, which potentiates 5-FU cytotoxic effects by stabilizing the drug-enzyme complex. Oxaliplatin is a second drug widely used on CRC, and it acts by binding to the DNA after its spontaneous biotransformation. Finally, irinotecan is a prodrug which is activated to its active metabolite called SN-38. This drug causes the inhibition of topoisomerase-I, leading to pro-apoptotic genetic damage [16].

These drugs are often combined in therapy regiments specific for different CRC stages: FOLFOX (5-FU/leucovorin + oxaliplatin) is used for stage III CRC; conversely, FOLFIRI (5-FU/leucovorin + irinotecan) or 5-FU/leucovorin + oxaliplatin and irinotecan (FOLFIRINOX) are used for stage IV. As many other chemotherapeutic agents, these too can cause a series of heavy side effects including nausea and vomiting, alopecia, neutropenia, and fatigue. The use of chemotherapeutic combinations allows for the decrease in the administered dose of each single drug and minimizes the chance of drug resistance by tumor cells.

More recently, targeted therapies against specific molecular targets overexpressed, or aberrantly expressed, by CRC are available. The main strategies rely on the use of small molecules (the so-called “-inibs” such as regorafenib [17]) or antibodies (e.g., cetuximab and panitumumab against EGFR). Bevacizumab and ramucirumab are similarly antibodies against VEGFR-1 and VEGFR-2. Ziv-aflibercept is a biotechnological drug composed of fragments of the VEGFR-1 and 2, fused with the Fc of human IgG1 that acts as a decoy receptor to block the action of VEGF. Today, the use of therapeutic monoclonal antibodies in combination with traditional chemotherapy is considered a staple in many adjuvant and neoadjuvant antitumor therapies, including CRC.

## 2. Nanotechnology in Colorectal Cancer Diagnosis and Therapy

In the last decades, nanotechnology gave a significant contribution to cancer detection, imaging, and therapy, as demonstrated by the large body of study for the development of nanobiosensors [18] and the presence of several FDA approved commercially available nanoparticles-based formulations. Nanotechnology still holds great promise for future improvements, with many nanovector-based formulations actually under evaluation in clinical trials [19].

Nanovectors are defined as nanoparticles with a diameter loosely between 1 and 100 nm in at least one dimension “capable of carrying and delivering one or more bioactive molecules, including therapeutic agents and imaging contrast enhancer” [20].

The rationale behind the use of nanotechnology in oncology (and in many other fields) lies in the unique feature that nano-scale materials offer as defined in 2000 by the US National Nanotechnology Initiative [21]. For instance, incorporation of nanomaterials in the traditional diagnostic assays may facilitate the overcoming of some technical difficulties, such as multiplexed analyte detection, very high sensitivity, and reproducible signal amplification for the quantification of biomarkers present at ultra-low concentration in body fluids [18]. In the frame of cancer therapy, the most employed approach is the use of intravenously administered nanovectors in order to improve the pharmacokinetic behaviour of otherwise problematic drugs. In particular, the encapsulation of consolidated or novel chemotherapeutics into nanoparticles improves their apparent solubility and ameliorates their biodistribution towards the tumor, avoiding off-target toxic effects. This contributes *de facto* to increase drugs therapeutic index and half-life, thus reducing their side effects while increasing their efficacy. Furthermore, since nanovectors are internalized by tumor cells by either endocytosis or macropinocytosis (and through phagocytosis by macrophages and dendritic cells), the encapsulation of chemotherapeutic drugs within these particulates can help them to bypass the multidrug resistance (MDR) pumps present on the cell membrane, which are responsible for the extrusion of free drugs that penetrate the cell by diffusion. Nanoparticles encapsulation is also of great benefit for biotechnological drugs (e.g., peptides, proteins, and oligonucleotides) that would otherwise be quickly degraded after systemic administration or for diagnostic molecules that would otherwise lack tissue specificity or be too toxic.

The same concepts of pharmacokinetic improvement can also be applied to diagnostic nanovectors, in order to deliver a higher amount of labelling molecules to CRC, avoiding their toxic effects and improving their sensitivity.

Nanoparticles should be carefully designed in order to finely tune their performances *in vivo*. In particular, three main aspects deserve considerations: (1) the NPs targeting mechanisms, (2) the NPs biodistribution and excretion *in vivo*, and (3) the NPs intrinsic chemical-physical properties.

### 2.1. NPs Targeting Mechanisms

This nanoparticle design philosophy relies on two complementary strategies:

- Passive targeting: this design relies on the interaction between the physiochemical features of the nanovector and the tumor microenvironment.
- The most well-known model of passive targeting is the enhanced permeability and retention effect (EPR effect), where nanoparticles of <200 nm diameter are able to extravasate from the systemic circulation through the *fenestrae* present in the defective solid tumor neovasculature and are retained by the tissue thanks to the dysfunction of lymphatic drainage [22].
- Other passive targeting designs include the use of pH sensitive [23], temperature sensitive [24], redox sensitive [25], or enzyme sensitive materials which can modulate drug release under pathology-specific conditions.
- Active targeting: this more recent blueprint relies on the knowledge of disease-specific molecular markers. These molecules are often membrane receptors which are overexpressed or exclusively expressed on the tumor cells. Therefore, functionalizing nanovectors with ligands for such markers would increase their targeting capabilities against the cells expressing the marker.

In CRC, the main molecular markers available for active targeting are the carcinoembrionic antigen (CEA) [26], the folate receptor alpha (FR*α*), and the epidermal growth factor receptor (EGFR and HER2) present onto the tumor cells. Additionally, possible targets against tumor neovasculature may be used that includes vascular endothelium growth factor receptor (VEGFR) and the integrin receptors [27].

The ligands for these targets include antibodies (e.g., cetuximab against EGFR and bevacizumab against VEGFR), peptides (e.g., the RGD peptide against the integrin receptor), and small natural molecules (e.g., folic acid against FR*α*), among others. Furthermore, since many solid tumours present often an unfavourable higher oncotic pressure than normal tissue, unfavourable to the extravasation of molecules (especially macromolecules and nanoparticles), the use of active targeting could improve tissue penetration and retention.

These molecular tags are also extremely relevant for the development of new *in vivo* diagnostic nanovectors for the detection and imaging of tumours.

### 2.2. NPs Biodistribution and Excretion *In Vivo*

The tuning of nanovector biodistribution requires also the avoidance of quick clearance from the organism. This is achieved by specific strategies, including

- Modulation of nanoparticle size: using very small nanoparticles can lead to their quick renal clearance by glomerular filtration. *Vice versa*, the use of large particles can induce their quick clearance by the mononuclear phagocyte system (MPS) of phagocytic cells present in the lungs, liver, and spleen, substantially reducing the amount of nanovector reaching the target tissue [28].
- Tuning of the nanovector surface features: it is well established that, after injection, highly positively or negatively charged, as well as hydrophobic nanoparticles interact with the surface of a wide range of endogen molecules. This phenomenon is often referred at as the “protein corona” formation. The rate, amount, and composition of this protein coating is critical in determining the fate of the nanosystem. In particular, numerous interactions with immunoglobulins and complement proteins can lead to the nanoparticle opsonisation and a quick MPS-mediated clearance. These interactions can also induce nanoparticle agglomeration, leading to serious side effects such as embolism, or they can cause hypersensitivity reactions [29].

In order to increase the nanovector circulation time and achieve better colloidal stability, the surface of nanoparticles should be overall neutrally or slightly negatively charged and be hydrophilic. This is achieved by coating the nanosystems with hydrophilic polymers, polyethylene glycol (PEG) *in primis*, which are capable to shield the nanoparticle surface from the external environment, slowing down the formation of the protein corona. However, this approach is not free from issues, since even “stealthy systems” can be opsonized to some extent and can induce the production of antipolymer antibodies after multiple administrations [30].

In the last years, the paradigm of nanoparticle design was revolutionized by the concept of biomimicry. This highly innovative philosophy relies on the use of biological molecules that bestow the nanovector with the same surface proprieties of circulating cells using autologous or syngeneic cell components as source material. This approach is able to recapitulate the biological complexity of the cell-membrane composition following a top-down strategy instead of the more traditional bottom-up chemical synthesis of nanovectors.

### 2.3. Chemical-Physical Properties of NPs

The capabilities of nanosized materials go well beyond the ones of simple drug or diagnostic agent's carriers. In fact, the intrinsic features of common materials can radically change at this scale, opening new horizons for the application of nanotechnology in oncology. In this context, since the use of nanovectors allows for the combination of drug delivery and the implementation of physical therapies such as thermal ablation, often the term “therapeutic payload” is used to describe the therapeutic action of the nanosystems in study. Furthermore, the combination of both therapeutic and diagnostic proprieties of nanovectors sparked the use of the term “theranostic” to define these novel multitasking platforms.

A clear example of this principle is metal-based nanoparticles. Superparamagnetic iron oxide nanoparticles (SPIONs) are characterized by high responsiveness to magnetic fields that is absent in the bulk material. This property makes possible their local accumulation by external application of localized magnetic fields. Because of their physical properties, SPIONs can also be used to thermally ablate tumours by the exposition to quickly alternating fields, causing heating of the nanoparticle surroundings. Finally, SPIONs may work as contrast agents for MRI. These particles are also by their own nature highly biocompatible and biodegradable, making them good candidates for therapeutic and diagnostic applications [31].

Another fascinating nanosized material is gold. Gold nanoparticles (AuNPs) are easy to synthesize, have relatively low toxicity, and can be easily functionalized by using thiolated molecules and are characterized by a peculiar surface electron oscillation known as surface plasmon resonance (SPR). This resonance occurs only at specific light wavelengths and can be modulated by nanoparticle size, shape, and proximity to each other allowing for its fine tuning. Therefore, it is possible to produce diagnostic gold-nanoparticles-based sensors able to change light wavelength absorbance upon NP agglomeration [32].

It is also possible to use the high light absorbance to achieve light-induced ablation of the tumor by reactive oxygen species (ROSs) production (photodynamic therapy, PDT), especially when using gold nanorods, nanoshells, or nanocages able to absorb near-infrared light, which has deeper tissue penetration than visible wavelengths.

Other applications of AuNP formulations include radiotherapy enhancements, X-ray contrast agents, and surface-enhanced Raman spectroscopy (SERS) enablers [33].

Despite AuNPs being known for decades, their relevance as nanovectors to this day is proven by recent studies [34–36].

**Figure 1:**
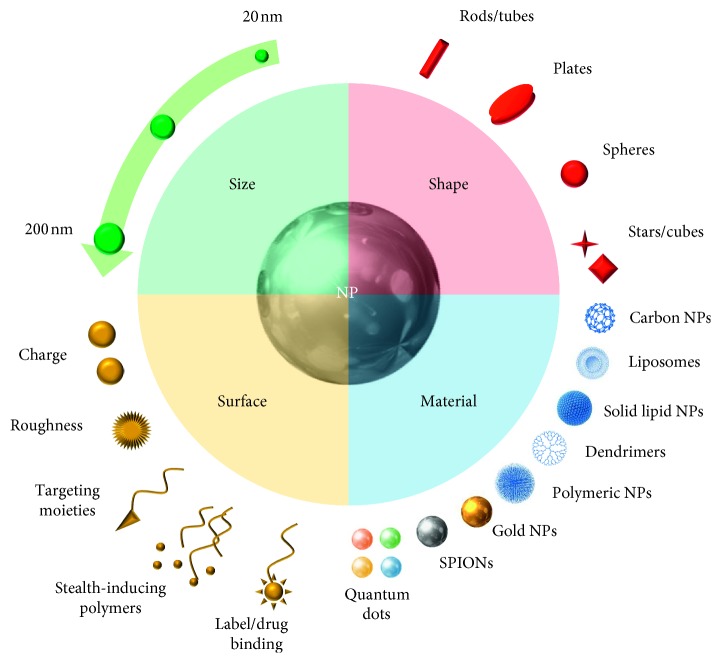
Schematic representation of the different features and design options considered during the formulation of nanovectors.

Furthermore, efforts are still being made in order to develop innovative nanomaterials with improved characteristics and versatility to potentiate radiotherapy and PDT, such as copper-cysteamine (Cu-Cy) nanoparticles [37]. An overview of all the possible proprieties of NPs for theranostic application is schematically represented in Figure 1.

The chemical engineering of these materials and the formulative techniques employed in the production of different nanovectors are extremely heterogeneous and dependent on the material in study and range from synthetic chemistry to biotechnology. This wide field deserves a stand-alone discussion. Since the aim of this review is to offer an overview of the latest design approaches and the new progresses and trends in nanoparticles-based theranostic applications against CRC, the reader interested in nanoparticle formulations is directed to exhaustive reviews focused on different materials [38–41].

Many drug delivery and diagnostic formulations have been designed for enteric administration, either through oral administration or during endoscopy using the endoscopic probe itself. However, the biological environment that these formulations interact with is much different from the one encountered by parenterally administered nanoparticles.

The gastrointestinal (GIT) lumen is in fact extremely complex. First and foremost, the pH and the epithelial composition vary a lot along the different GIT segments, as does the microbiota composition. This layout is even more complicated by possible alterations of all these parameters by pathological states. All these variables and harsh conditions require the use of highly selective and stable formulations capable of targeting the tumor tissue, adhering to it, while avoiding absorption and off-target effects.

Very importantly, the formulation of particle-based systems for enteric administration is not constrained in terms of size as the parenteral nanovectors, since the intestinal lumen does not require stringent size conditions to avoid embolism. On the contrary, many enteric platforms are deliberately designed with larger diameters in order to prevent the intestinal absorption of these particles, thus avoiding possible systemic toxicity.

However, the large size of these formulations exceed the upper limit that is usually set for the definition of “nanoparticles” (200 nm in diameter) and are often referred at as microparticles. Thus, these formulations are not included in the present review.

### 2.4. Nanoparticles for CRC Detection and Imaging

#### 2.4.1. Detection of the Primary Tumour

*(1) Nanovectors for Intravenous Administration*. In the last decades, the surgical treatment of CRC has remained a therapeutic staple, and huge efforts have been made to make it as least invasive and as safe as possible. In this regard, the use of laparoscopic procedures has been a great leap forward. However, in the majority of cases, the removal of CRC tissue remains quite extensive and includes large portions of intestine, vasculature, and lymphatic system, such as proximal lymph nodes in order to avoid tumor relapse and metastasis [42]. Hence, better tumor imaging could lead to smaller resections without increasing the risk of relapse.

With this in mind, many advancements have been achieved in the improvement of optical devices, but there is still a need for proper probes able to synergize with these systems. Nanomedicine has the potential to work in tandem with endoscopic techniques to achieve more precise and personalized surgery. Furthermore, the endoscopic analysis to this date relies on morphological analysis of the intestinal epithelium looking for alterations and lesions. However, this technique does not allow for the assessment of the tumor on a molecular level. The use of NPs able to detect the presence of specific soluble molecules or receptor which are characteristic for CRC can substantially improve the diagnosis based on real-time endoscopy.

One of these formulations has recently been produced by Tiernan et al. [43]. These nanoparticles are composed of a core of silica doped with NIR fluorophore NIR664 which allows tumor visualization by high tissue penetration of the stimulating light. These particles were then functionalized with a humanized anti-CEA antibody through the use of polyamidoamine (PAMAM) dendrimers. This formulation showed higher *in vitro* accumulation in CRC cell cultures compared to the untargeted NPs. In *in vivo* CRC xenografted mice, these nanoparticles showed similar liver accumulation to the untargeted formulation but with much higher tumor-associated fluorescence, making this nanosystems a good candidate for clinical translation in intra-operative CRC detection.

*(2) Nanovectors for Local Administration*. Since the surgical removal of early-stage CRC is considered curative, the employment of better techniques to detect polyps and adenomas could result in a swift and efficacious surgical removal of the affected tissue without the use of radiotherapy nor chemotherapy.

With this in mind, Chen et al. developed an anti *α*-L-fucose actively targeted PEGylated MSNs-based formulation loaded with fluorescein (FITC) for optical visualization of early tumor lesions by endoscopy after enteric administration [44]. *α*-L-fucose is a sugar overexpressed on the surface of CRC adenomas and early neoplasms and has been successfully targeted using Ulex europeaus agglutinin-1 (UEA1). Also, MSNs can efficiently load fluorescent molecules without causing any fluorescence quenching, and PEGylation demonstrated to increase mucus stability, adhesion, and penetration of nanoparticles after enteric administration.

These UEA1-targeted nanoparticles showed good stability in intestinal mucus and were able to target *α*-L-fucose positive cells in a UEA1-dependent specific fashion. Furthermore, this system was also able to evidence *α*-L-fucose positive foci in *ex vivo* murine intestine samples, as they were *in vivo* in DSS/AOM-induced tumor-bearing mice as evidence by endomicroscopy analysis after topical administration of NPs.

The intraluminal administration of NPs-based formulations can substantially improve the sensitivity and information provided from a well-established endoscopic technique. NPs can enhance the detection of small and early neoplastic foci, thus providing earlier detection and a better prognosis. Furthermore, the use of active targeting moieties provides important information on the molecules feature of the tumor in real time.

A recent study from Kolitz-Domb et al. tested NPs composed of L-glutamic acid, L-phenylalanine, and poly (L-lactic acid) (P(EF-PLLA)). These nanoparticles were loaded with the NIR label indocyanine green (ICG) for early CRC adenocarcinoma detection. NIR fluorophores provide an emission that can penetrate tissues and is not affected from tissue absorption. This formulation demonstrated good colloidal stability over long periods of storage, provided good photostability for ICG, and retained the fluorophore. After IV administration, they demonstrated a wide biodistribution among different organs and quick clearance with no evident toxic effects, thanks to their good biodegradability. Furthermore, after this NP functionalization with anti-CEA antibodies provided good targeting capabilities to P(EF-PLLA) NPs in a tumor engrafted chicken embryo chorioallantoic membrane (CAM) model. Finally, P(EF-PLLA) were administered through the colon into a CRC orthotopic murine model, demonstrating good targeting and imaging enhancement of the tumor foci [45].

An analogous study investigated the use of NIRF label IL-783 conjugated to a N-(2-hydroxypropyl)methacrylamide (HPMA) copolymer based nanovector, actively targeted against the early CRC marker under-glycosylated mucin-1 antigen (uMUC-1) using the synthetic peptide EPPT1. This formulation was able to bind uMUC-1-expressing cell cultures in direct proportion to antigen expression levels. Furthermore, these conjugates were also able to bind to CRC patient samples compared to healthy tissue. Furthermore, when administered intraluminally into an orthotopic CRC mouse model, this conjugate was able to bind significantly the tumor more than the nontargeted formulation after colon harvesting [46].

**Figure 2:**
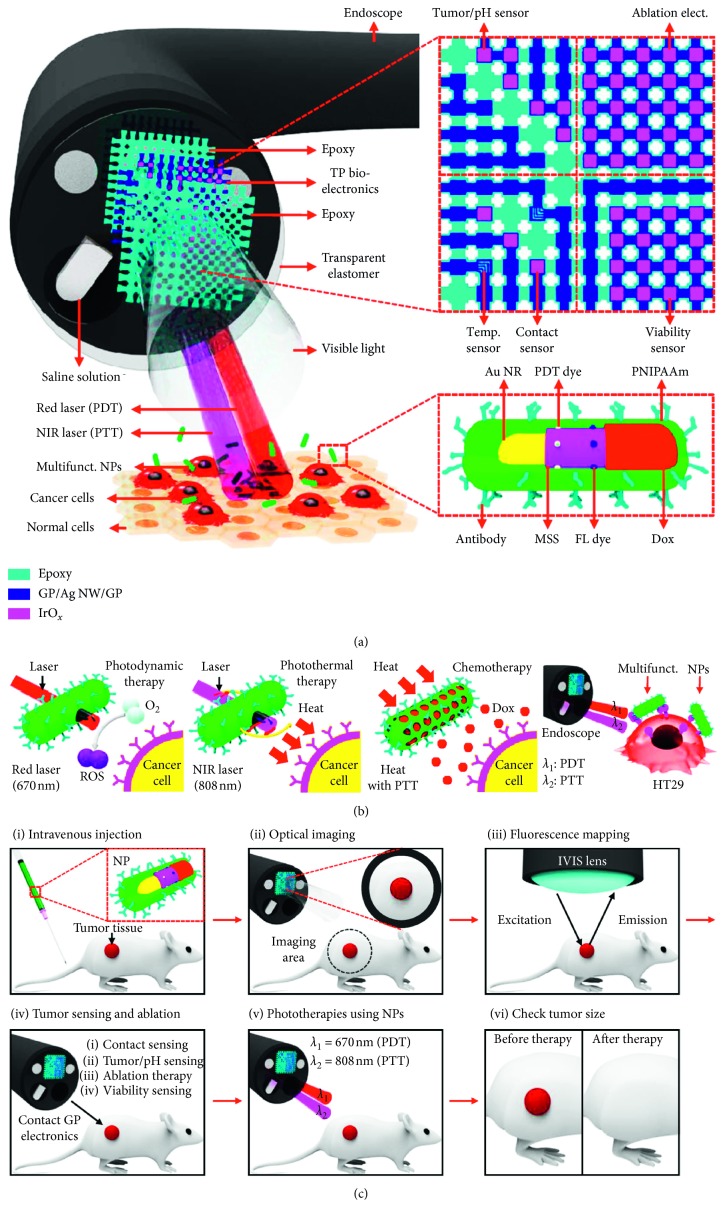
(a) Design strategy and mode of use for the endoscopic device and of the theranostic nanoparticles employed. (b) Schematic representation of the different theranostic approaches enabled by the endoscope/nanoparticle platform (from left to right): photodynamic therapy, photothermal therapy, chemotherapy, and synergetic effect of multimodal phototherapies under pulsed laser irradiation (c) Graphical concept of tumor treatment using the endoscope/nanoparticle platform. Figures from reference [47] reprinted with modifications.

A very interesting and innovative application of core-shell gold nanorods was their testing in combination with endoscopy (Figure 2). In particular, the study employed gold nanorods, capable of absorbing NIR right and obtaining photothermal effect, covered with a layer of mesoporous silica loaded with doxorubicin as a chemotherapeutic agent (released by the silica only upon NIR irradiation), a fluorescent dye for in vivo imaging, and a photosensitizer molecule to enable photodynamic treatment capable of producing ROSs upon irraidation by a differed red laser. Finally, this system was coated with a polyacrylamide copolymer and functionalized with active targeting with cetuximab to target *in vitro* and *in vivo* tumor cells. This complex and multifunctional nanoparticles were used in combination with an *ad hoc* endoscopic probe. The protocol consisted in administering the nanoparticles intravenously and letting them achieve tumor accumulation. The probe was inserted into the colon, and the fluorescent dye on the particles was used in order to detect vey precisely the localization of cancer cells. This mapping in turn makes possible the local ablation of tumor cells using the probe itself and then verifying the extent of the lesions using different probe sensors for pH and temperature upon contact with the tissue. The further application of the red and NIR laser finally triggered the nanoparticle activation and ROSs production and heat production, respectively, with consequent local release of doxorubicin. This combined multimodal therapy showed to be particularly efficient in mice planted with HT-29 cells. The nanoparticles were previously tested *in vitro* for different proprieties and showed good tolerability and stability, no significant leakage of doxorubicin before activation, and a dependence in their PDT and PTT effects from concentration, irradiation time, and potency. The combination of different treatments showed a significant increase respectively to single chemo, PDD or PTT [47].

In a similar experiment, Kim et al. fabricated 350 nm NPs for multiplex CRC imaging using endoscopy [48]. These nanoparticles were composed of s silica core coated with SERS-labelled Ag nanoparticles: this structure was further coated with a mesoporous silica layer functionalized with fluorescent labels and specific anti-EGFR or anti-VEGF antibodies (F-SERS-A and F-SERS-B, respectively). This formulation demonstrated remarkable chemical stability in time and high photostability upon irradiation. These distinctly targeted formulations allow for the fluorescent-SERS multimodal detection of CRC markers (EGFR) and characterization of the tumor microenvironment (VEGF). NPs were administered by topical spray onto the tumor site in orthotopic xenograft murine CRC models. The treatment allowed for efficient localization of the tumor through fluorescence (using the same fluorophore in both formulations) and EGFR/VEGF detection through the use of distinct SERS labels. This system did not show any systemic toxicity thanks to the topical administration route and was able to detect small tumours with a signal proportional to cell density and to the dose administered. The use of different antibodies as active targeting moieties makes these NPs quite versatile in their application to different tumours.

These formulations are both outstanding examples of how NPs-based tumor detection systems can be implemented in conjunction with established diagnostic techniques to improve their sensitivity and the molecular profiling of tumours in real time.

#### 2.4.2. Detection of Sentinel Lymph Nodes

A pressing issue in CRC surgical removal is the proper tracking of the tumor sentinel lymph nodes (SLNs), which are the ones more important to remove in early-stage CRC in order to prevent or assess the presence of lymphatic metastases. Since to this day, there is no primary tumor feature for the prediction of metastasis, the histological analysis of lymph nodes is essential for proper tumor staging, therapy choice, and prognosis [49].

At the moment, the most common ways to locate SLNs in CRC rely on the use of locally injected soluble dyes or isotopes, which are able to be drained from the submucosa into the lymphatic system, labelling SLNs; however, these soluble dyes diffuse very quickly beyond the SLNs and must be administered intra-operatively. Furthermore, the use of radioactive dyes with Geiger counters raises some concerns regarding their safety. Recently, some preliminary studies have employed indocyanine, a fluorescent dye that can be stimulated via infrared electronic endoscopy as a SLN tracker, with an accuracy of 94%.

In a recent multicentre study on 74 early-stage CRC patients, 1 ml of 150 nm carbon nanoparticles (CNPs) were injected in the submucosa during endoscopy 24 h before surgery. The rationale in the use of CNPs relies in their very peculiar biodistribution: differently from the soluble molecules described above, these NPs are phagocytized by resident macrophages and then carried to the closest lymph nodes, dying them in black. These nanoparticles are too large to extravasate in the vessels, are retained in the SLNs for more than 24 h, and do not require any concomitant instrument for lymph node detection.

The use of CPNs was also demonstrated to be useful to improve the nodal staging of CRC. In a study from Wang et al., submucosal peritumour administration of CNPs as LN markers in rectal cancer patients allowed for much quicker surgical removal of much more LNs compared to the CNPs free control group. The extraction of more lymph nodes in turn allowed for a more accurate metastatic LN detection, an assessment that is often underestimated by clinicians because of the insufficient LN pool retrieved from surgery and used as sample. This can lead to better prognosis and better postoperative therapeutic choices, substantially improving the treatment efficacy [50].

#### 2.4.3. Detection of CRC Metastasis

The recent employment of biologic components for the synthesis of NPs paved the way for many innovative formulations. One of such nanovectors is NPs composed of a three-way junction packaging RNA (3WJ-pRNA) derived from bacteriophage phi29. These NPs are able to self-assemble in a trimetric thermodynamically stable structure, which is not affected by nucleases, and is biodegradable and nonimmunogenic. Furthermore, 3WJ-pRNA NPs are easy to functionalize with targeting moieties, labelling agents, and drugs.

A recent work from Rychahou et al. used 3WJ-pRNA NPs actively targeted with folic acid and labelled with the NIR-detectable fluorophore Alexa fluor-647 as detecting system for the liver and lung FR*α* positive CRC metastasis. These nanoparticles demonstrated remarkable targeting of FR*α* positive cells *in vitro* and *in vivo*. These nanoparticles did not accumulate in healthy, metastasis free liver, and lungs parenchyma, avoiding MPS clearance, and were eliminated quickly through renal clearance. The versatility and novelty of this platform makes it quite promising for both imaging and therapy of CRC [51].

#### 2.4.4. Nanoparticles for CRC Therapy

Nanomedicine offers the opportunity to re-evaluate the employment of established drugs approved for disparate applications in cancer treatment by improving their apparent solubility and their biodistribution.

One recent example of this principle is provided by Bhattacharyya et al. who chose nicosamide (NIC) as anti-CRC drug thanks to its ability to down-regulate the Wnt/*β*-catenin pathway. This group formulated self-assembling micelles composed of a hydrophilic chimeric elastin-like polypeptide conjugated to multiple molecules of the hydrophobic drug nicosamide to the ends of the peptide chains. This led to the spontaneous self-assembly of tubular micelles containing in their core drug. This formulation was able to inhibit Wnt pathway and reduce tumor cells viability *in vitro*. Furthermore, these nanoparticles were able also to increase nicosamide plasmatic half-life and significantly reduce tumor growth in HCT116-xenografted nude mice, slightly increasing mice survival [52].

#### 2.4.5. Nanovectors for Drug Delivery against CRC

Despite the long history of monoclonal antibodies (mABs) as active targeting moieties for nanovectors, mABs are not free from possible pitfalls, including instability of secondary and tertiary structure (folding and agglomeration), difficulties in chemical modification (site-specificity of modification, restrictions in the use of organic solvents, or high temperatures), and antigenicity issues. These limitations prompted the research for new, small, nonantigenic and easily functionalized molecules. An elegant solution was found in the use of aptamers (APts), small engineered DNA or RNA oligonucleotides capable of folding into specific conformations with high affinity for proteins.

These novel targeting agents have been used recently for the functionalization of nanovectors, as reported in a study by Li et al. [53]. In this investigation, mesoporous silica nanoparticles (MSNs) were loaded with maytansine (MDI), a naturally derived cytostatic drug which is able to disrupt microtubules polymerization with much higher efficacy than vinca alkaloids. These nanoparticles were then covered with a pH-dependent polydopamine (PDA) coating (allowing the drug release upon the acidic conditions of the cellular lysosomes) and functionalized with anti-EpCAM APts functionalized PEG, allowing active targeting and RES avoidance, respectively. EpCAM is an adhesion molecule overexpressed in many quickly proliferating tumours, including CRC.

This nanovector demonstrated *in vitro* SW480 efficient tumor cell uptake, cytostatic effect, and apoptosis when compared with normal epithelial NCM460 cells. Furthermore, the *in vivo* biodistribution experiments highlighted an increased accumulation of the actively targeted formulation in the tumor than in the liver, kidneys, and lungs when compared with the nontargeted nanoparticles, with significant reduction of tumor growth rate.

For many years now, the development of nanoparticles has used viruses as an ideal paradigm of nanovectors design. In fact, viruses are able to efficiently move into systemic circulation, avoid quick clearance from the immune system, and efficiently target specific tissues, efficiently delivering their cargo. These are all critical features needed for proper nanovector action.

Henceforth, biotechnology applied to the production of virus-like nanoparticles, led to the development of innovative nanovectors for CRC composed of the same building blocks of naturally occurring viruses.

One of such formulations is composed of modified Hepatitis B core antigen (HBcAg), which is capable of self-assembling in 35 nm nanoparticles [54]. This peptide was further modified via a pentadecapeptide nanoglue linker to present folic acid (FA) as an active targeting moiety against FR*α*-expressing tumor cells. In order to load the chemotherapeutic doxorubicin in these nanovectors, the drug was electrostatically complexed with polyacrilic acid, forming a pH-reversible bond. This system showed pH dependent release of the drug and a FA-dependent uptake and cytotoxicity on CRC HT-29 and Caco-2 cell cultures, which was much higher than in normal colorectal CCD-112 cell lines.

This system shows how the use of biological components together with biochemical modification can allow for quick formulation of novel nanovectors.

The use of multiple and realistic preclinical animal models is critical for the proper understanding of a formulation true therapeutic potential. This requirement was underlined in a study from Li et al. who synthesized dextran-based NPs containing covalently conjugated doxorubicin and cisplatin as a crosslinker (Dex-SA-DOX-CDDP NPs). This formulation demonstrated good stability and similar cytotoxicity to the free drugs. Dex-SA-DOX-CDDP NPs were tested also on a widely used subcutaneous murine model of CRC and on an *N*,*N*′-Dimethylhydrazine dihydrochloride-induced orthotopic model of CRC. This latter model is considered to be more similar to real CRC since it is orthotopic and develops in a multistage manner similar to the clinical CRC manifestations. In both models, NPs were able to decrease tumor growth with reduced liver and cardiac toxicity compared to the free drugs, causing tumor necrosis and ultimately increasing the subjects' survival [55].

The use of red blood cell membranes (RBCms) to coat NPs was one of the first biomimetic strategies adopted to avoid MPC clearance of IV injected NPs, since RBCs are by their own nature long circulating cells. The long RBCs plasmatic half-life is mediated by surface “self” markers such as CD45.

Recently gambogic acid (GA), a hydrophobic cytotoxic natural molecule, was loaded into RBCms-coated PLGA nanoparticles prepared by co-extrusion of isolated RBCms and GA-loaded PLGA nanoparticles assembled by an emulsion evaporation technique. These nanoparticles demonstrated good stability, drug loading, slow drug release, and good biocompatibility. Furthermore, they showed 48h *in vitro* cytotoxicity on SW480 cells comparable to free GA, but at the same time, increased reduction in tumor size and survival *in vivo* compared with free GA in male BALB/c mice subcutaneously implanted with SW480 cells [56].

However, since the use of RBCms do not provide any active targeting capability, the same group created a recombinant protein capable of binding the integrin receptors as well as EGFR (anti-EGFR-iRGD protein) purified from transfected *E. coli*. The use of a double active targeting should synergistically enhance CRC passive targeting. Henceforth, a new formulation of anti-EGFR-iRGD-RBCms-PLGA NPs loaded with gambogic acid was formulated.

This system demonstrated much higher HT-29 cells spheroids penetration than the nontargeted formulation *in vitro*, and when loaded with GA, had similar cytotoxicity profile compared with the free drug. Also in *in vivo* experiments on Caco-2 bearing mice, the GA-loaded actively targeted formulation showed high tumor targeting and substantial RES avoidance. Furthermore, this formulation was able to quench tumor growth substantially and without affecting mice health, as confirmed by the normal weight and survival increase displayed by the GA-loaded NPs treated group [57].

In the recent years, the advancements in tumor biology evidenced the importance of the so-called “cancer stem cells” (CSCs) subpopulations in cancer maintenance, resistance to therapy, relapse, and unfavourable prognosis. This minority of tumor cell population is characterized by low differentiation, high resistance to chemotherapeutics and pluripotency, being particularly elusive, and capable to shift between dormant drug resistant condition and tumor cells, *de facto* inducing tumor relapse [58].

In this context, the discovery of new membrane molecular markers for CSCs could prime the development of novel, actively targeted nanovectors specifically directed against this cell subpopulation. In particular, in a recent paper from Ning et al., polymer-based micelles of poly (ethylene glicol)-co-poly (*ε*-caprolactone) and maleimide-poly (ethylene glicol)-co-poly (*ε*-caprolactone) functionalized with an anti-CD133 antibody and loaded with SN-38 have been developed. CD133 is a cholesterol binding protein that is overexpressed in many CSCs in different tumours, including CRC, and is associated with higher tissue invasion and metastasis formation. This system demonstrated slow drug release and good stability. The *in vitro* testing demonstrated that their uptake and cytotoxicity are dependent on anti-CD133 mAB active targeting and is proportional to CD133 expression in different cell lines (especially on CD133-high HCT116 and SW620). The *in vivo* testing showed substantial tumor growth reduction after administration, and the immunohistochemical analysis revealed a relevant reduction of CD133 + cells in animals implanted with SW620 subcutaneously [59]. This system therefore demonstrated great innovative potential in CSCs targeting and elimination.

It is now well-known that tumor induction and progression is a complex interplay of different genetic and environmental factors. In particular, the tumor microenvironment has been established to be a critical determinant in tumor development, invasion, even affecting the therapeutic outcomes. The tumor microenvironment is composed by immune cells, neovasculature, stromal cells, and a wide array of proteins known as the extracellular matrix (ECM). These two latter components are able to cause substantial tissue remodelling under pathologic conditions, and many tumours including CRC have a poorer prognosis when their microenvironment appears stiffer and fibrotic (a condition defined as “desmoplastic”), with increased mechanic stress and reduced blood perfusion caused by vessel compression [13].

Therefore, the normalization of tumor microenvironment is nowadays considered a novel potential therapeutic target. Recently, a nanosystem developed with this aim was based on 15 nm AuNPs loaded with cisplatin [36]. In the *in vitro* studies, AuNPs were able to reduce the tumor cells production of pro-fibrotic factors such as TGF-*β*, CTGF, and VEGF. These nanoparticles were able to reach implanted CRC tumor cells subcutaneously in mice after IV administration. Once reached the tumor site, these nonloaded NPs interacted with ECM molecules (and collagen I in particular) causing their destabilization and overall decrease, and also with tumor-associated fibroblasts, reducing their production of ECM components, and their proliferation. This led to decreased solid stress and increased perfusion by vessel decompression. However, these nanoparticles were not able to reduce tumours cells viability neither *in vitro* nor *in vivo*. After their loading with cisplatin, the AuNPs were able to increase its delivery to the tumor, reducing significantly tumor growth.

Solid tumours are often characterized by microenvironmental acidosis, often caused by rapid cell growth not properly sustained by adequate blood flow, causing a metabolic shift of the tumor cells towards anaerobiosis and therefore the production of lactic acid. This tumor feature too has been exploited as passive targeting strategy in order to achieve tumor-specific drug release.

A very recent work by Zhao et al. was focused on the formulation of chitosan nanoparticles loaded by covalent bonding with the ROSs-inducing molecule cinnamaldehyde (CA) and 10-hydroxy-camptothecin (HCPT). These particles were able to release the drugs in a pH-dependent fashion, to cause ER-stress, selective tumor cells apoptosis by ROSs production and prevent colony formation and wound healing of tumor cell lines *in vitro*. Furthermore, the encapsulated drug showed increased plasmatic half-life *in vivo* and decrease renal toxicity of the drugs, increasing tumor accumulation and significantly reducing tumor growth [60].

Another interesting aspect of tumor microenvironment is the production of tissue remodelling enzymes that enhance further tumor invasion and metastatic potential. In CRC, it has been evidenced an increase in the levels of proteolytic enzymes, in particular the matrix metalloproteinase-2 and metalloproteinase-9 (MMP-2 and MMP-9). As previously mentioned, these enzymes can be a potential mean of passive targeting by the engineering of nanovectors that release their cargo upon MMPs-mediated hydrolysis [61].

Following this principle, Shi et al. formulated synthetic self-assembling nanoparticles composed of PEG-peptide diblock copolymer (PPDC), in which the hydrophobic peptide is sensitive to MMP-2 cleavage. These nanoparticle displayed MMP-dependent *in vitro* drug release of the anti-angiogeneic drug sorafenib and of the model chemotherapeutic CPT. Furthermore, this system was capable of causing cell toxicity in CRC cell lines spheroids expressing MMP-2 (HT-29). This system was also able to down-regulate angiogeneic signalling through sorafenib and CPT synergistic action, inducing tumor cell apoptosis. This formulation showed good EPR-mediated targeting of tumours in HT-29 bearing mice, reducing the vascularization of the tumor and causing tumor necrosis, *de facto* stopping tumor growth when compared with the free drug combinatorial treatment [62].

#### 2.4.6. Nanovectors for Gene Therapy against CRC

Innovative therapies with biotechnological molecules and gene therapy have revolutionized antitumor therapy in the last decades. Gene therapy is also considered a good alternative to direct cytokines administration in antitumor therapy in order to avoid the risk of serious systemic toxicity, and two main strategies have been employed so far to achieve *in vivo* transfection: the use of viral vectors or the use of nonviral nanoparticles.

This latter option allows avoiding the use of viral vectors which often raise concern regarding their efficiency and safety such as recombinant re-activation and oncogenicity. However, the delivery of oligonucleotides to tumours poses a lot of challenges regarding their stability and transfection efficiency *in vivo*, and the use of *ad hoc* designed nanovectors can satisfy the need for good tumor targeting, shielding of the oligonucleotide cargo from nucleases normally present in systemic circulation, and increased penetration of the cells by endocytosis, thus increasing the transfection efficiency.

Interleukins are strong pro-inflammatory molecules, with the potential to induce an antitumor immune response. However, their instability and serious toxicity upon systemic administration poses a serious hurdle to the development of cytokine-based therapies. A potential way to bypass this issue is to induce the expression of cytokines in the tumor tissue by selectively transfecting tumor cells. In this context, NPs-based formulations represent a promising solution.

With this in mind, Liu et al. developed a novel methyl-PEG-polylactide (mPEG-PLA) and 1,2,-dioleoyl-3-methylammonium-propane (DOTAP) in order to deliver an IL-12 plasmid (pIL-12). This formulation was able to yield an above 50% transfection efficiency on CRC Ct26 cell line, inducing the production of IL-12. Furthermore, the IL-12-containing supernatants from transfected cells were able to induce activation of mouse spleen-derived T-cells, inducing their proliferation and production of TNF*α* and IFN*γ*. Also, the supernatant of activated T-cells was able to quench tumor cell growth, suggesting that T-cell mediate the antitumor immune response of IL-12. This was confirmed by *in vivo* experiments on tumor-bearing mice, where the nanoparticles were able to induce IL-12 production which resulted in increased IFN*γ* and TNF*α* in the tumor milieu, thus tumor growth without any sign of systemic toxicity [63].

Similarly, a recent study focused on the delivery of a peculiar fusion plasmid containing DNA for IL-21 and the tumor antigen NKG2D using chitosan-based nanoparticles [64]. IL-21 is considered a highly pro-inflammatory cytokine expressed by a wide array of immune cells. This cytokine is able to induce CD8+ and NK T-cells activation, potentially leading to an antitumor immune response. NKG2D is an antigen not present in healthy tissue but expressed by virus-infected and tumor cells, and has good immunogenic potential. The expression of a KGG2D-IL21 fusion protein by transfected tumor cells is expected to trigger a strong antitumor immune reaction. This system was efficiently internalized by CRC cell lines and was also able to induce the secretion of KGG2D-IL21 in the cell supernatant as demonstrated by Western blot. Furthermore, the transfected cells were able to induce spleen-derived mononuclear cell activation in coculture, causing in turn inhibition of the transfected cell growth. When injected intramuscularly in normal mice, the NPs were able to induce a systemic increase in circulating IL-21 and increased substantially splenic CD4+ and CD8+ activated T-cells number. Injected IV in tumor-bearing mice, this formulation was able to accumulate into the tumor tissue. Finally, after reaching the tumor and transfecting cells, there was a substantial increase in the number of lymphocytes, NK, CD8+, and CD4+ activated cells, which in turn led to a significant decrease in tumor growth rate and an increase in mice survival.

Tumor cells themselves can be transfected in order to express pro-apoptotic molecules in order to induce their own death. This was achieved in a recent work by Li et al. [65]. In this study, polymeric dual-targeting nanoparticles able to target both CD44 and integrin *α*_v_*β*_3_ overexpressed by CRC cells (RRPH/PF_3_/pDNA) were loaded with a plasmid encoding for tumor necrosis factor-related apoptosis inducing ligand (TRAIL). This protein is able to bind to death receptors 4 and 6 (DR4 and DR5), which are often overexpressed in CRC. These nanoparticles were able to efficiently target CRC cell lines *in vitro*, which was inhibited by the presence of excess of free CD44 and integrin *α*_v_*β*_3_ (HA and RGD, respectively) and showed high transfection efficiency, delivering fluorescently-stained DNA to the cell nuclei. These nanovectors were also able to increase the expression of TRAIL by CRC cells, inducing increased levels of cleaved caspase-9, which in turn caused high levels of cell apoptosis *in vitro*. Furthermore RRPH/PF3/pDNA NPs were able to significantly reduce tumor weight and the number of tumor nodules in a murine model of CRC peritoneal metastasis.

The use of anti-miRNA ONTs is therefore believed to be a good candidate for the treatment of such tumours and could greatly benefit from encapsulation in nanosystems which can increase their biological stability and tumor delivery.

Following this principle, MSN-PDA nanoparticle formulation was synthesized, this time actively targeted through an antinucleolin aptamer (AS1411) and loaded with an anti-miR 155 ONT [66]. MiR-155 is a small interfering RNA (miRNA) overexpressed in many malignancies, including CRC, and is related to less differentiated tumours, higher stadiation, and drug resistance.

This system showed good stability and tolerability *per se* and was able to efficiently deliver anti-miR-155 to tumor cells cultures and subsequent miR155 silencing when compared to the nonactively targeted formulation. Furthermore, this nanovectors was able to deliver the cargo to SW480 tumours subcutaneously injected in nude mice, with relatively low accumulation in other organs, hence inhibiting tumor growth.

Furthermore, these NPs were able to display a synergistic activity when co-administered with 5-fluorouracil (5-FU). The silencing of miR155 was showed in fact to be related to decrease in the expression of the multidrug resistance-inducing Pgp, responsible for 5-FU lack of efficacy.

MiRNAs themselves can also be used as therapeutic agents in order to modulate tumor cells gene expression, and the use of NPs can substantially improve their biodistribution and increase their *in vivo* stability.

A recent application of this concept was also given by Wang et al. [67]. This group synthesized polymeric nanoparticles composed of self-assembling triblock co-polymers composed of PEG (to achieve stealthiness), poly-L-lysine (to complex ONTs), and poly-phenylalanine. These NPs were able to complex miRNA-139, an ONT involved in tumor growth and metastasis inhibition (miRNA-139-NPs). This formulation showed relevant toxicity on CRC cell lines only when loaded with miRNA-139. Furthermore, miRNA-139-NPs were able to substantially reduce tumor growth in both a CRC xenograft and in an orthotopic murine model, with no evident sign of toxicity, when compared with free miRNA-139. These NPs were also able to significantly increase survival and reduce metastasis formation.

This is truly a remarkable example of how both new therapeutic agents and established anticancer drugs can benefit from the application of nanomedicine.

The employment of gene therapy can be extended for the manipulation of the tumor microenvironment by modulating the gene expression of the surrounding cells that contributes to its growth and modulate the composition of the extracellular matrix. In a recent study, Marquez et al. focused on the treatment of liver sinuosoidal endothelilal cells (LSECs), which are considered major contributors to the development of CRC liver metastasis [68]. To achieve this, the author encapsulated miR-20a (selected through micro-array screening), as a viable cargo to restore the physiological gene expression profile of LSECs. This miRNA was encapsulated into chondroitin sulphate-functionalized nanoparticles (SP-OA-CS). These glycosylated nanoparticles are capable of active targeting towards LSECs through the mannose receptors and hyaluronan receptors [38, 39]. These nanoparticles were able to efficiently target and transfect isolated murine LSECs *in vitro* with a model EGFP plasmid and reduce their migration. Furthermore, these nanoparticles were able to significantly inhibit the expression of ARHGAP1 and E2F1, two proteins involved in CRC liver metastasis progression. This formulation was also able to target the LSECs *in vivo*, avoiding the uptake from local Kupffer phagocytic cells, reducing the number of metastatic liver foci and decreasing LSECs metastatic infiltration. This study demonstrates how NPs can be a useful tool for the testing of novel biological cargoes identified through genetic screening, underlining their translational potential in terms of versatility.

Activated hepatic stellate cells (aHSCs) are considered major mediators of liver fibrosis. Fibrosis is considered an important factor in the development of CRC liver metastasis, since this promotes the polarization of local macrophages towards a protumor M2 phenotype, induces T-cell anergy, and shields cancer cells from direct contact with immune cells through the increased stiffness of the extracellular matrix.

In a similar study, Hu et al. developed a novel approach for the treatment of CRC liver metastasis based on the modulation of the extracellular matrix in synergy with PD-L1 checkpoint blockade [69]. This group encapsulated in NPs a plasmid encoding for relaxin (RLN). Relaxin is a peptide which binds to the receptor RXFP1, thus reducing liver fibrosis. The nanovectors for this transfection are PEGylated lipid-calcium phosphate NPs, which are also functionalized with aminoethyl anisamide to actively target aHSCs and metastatic cells overexpressing its receptor Sig-1R. This formulation was able to efficiently target CRC and transfect CRC cell *in vitro*. Furthermore, these nanoparticles administered IV were able to accumulate in the liver, specifically in CRC and aHSCs, and to transfect them to express RLN. This transfection was related to decreased expression of protumor cytokines (TGF-*β*, FGF, PDGF, CCL2, CCL5, IL4, IL6, and IL10), decreased production of collagen and decreased levels of pSMAD2/3 (a transcription factor involved in fibrosis), while at the same time increasing the expression of pro-inflammatory cytokines such as IFN*γ* and IL12. RLN transfection also demonstrated remarkable synergy with PD-L1 checkpoint blockade, achieved by transfecting CRC cells and aHSCs with a plasmide encoding a PD-L1 trap peptide. This combined treatment increased substantially the number of CD8+, CD4+, and mature dendritic cells in the tumor milieu, while reducing the number of *T* regulatory cells and MDSC. All these microenvironment alterations translated in an almost complete hindrance of tumor growth and led to significantly increased survival.

#### 2.4.7. Nanovectors for Physical Therapy against CRC

The use of physical-based therapies, such as photothermal therapy (PTT), opened new exciting perspectives in cancer therapy. These strategies often rely on the use of sensitizing molecules, which often suffer from poor stability, solubility, or that are too systemically too toxic for a stand-alone administration. The use of nanovectors can help to solve all these issues, in the same way they improve chemotherapeutics pharmacokinetics.

Following this strategy, Zhang et al. recently formulated a novel combinatorial drug delivery system composed of silica-coated cerasomes loaded with doxorubicin and indocyanine green (Cy7) as PTT sensitizer and NIR fluorescent imaging molecule (Gly@Cy7-Si-DOX NPs). These spherical nanoparticles demonstrated excellent stability and relatively low toxicity *in vitro* and *in vivo* after efficient EPR-driven tumor accumulation and internalization by cells. Furthermore, their cytotoxic effect was only triggered under acidic condition, which cause doxorubicin release, and under NIR irradiation, which caused cell-damaging heat and destabilization of the internalized NPs, allowing drug release. These NPs were able to almost completely eradicate xenografted tumours *in vivo* without any relapse and improve survival. However, the majority of NPs accumulated in the liver and spleen, causing some histological alterations [70].

In a similar study, White et al. formulated iron oxide/gold hybrid nanoparticles functionalized with anti-MG1 antibodies on their surface linked through a PEG molecule (aMG1-PEG-HNPs). These nanoparticles are another example of multifunctional theranostic platform, since the iron core allows for MRI detection, and the gold coating allows for PTT upon 800 nm light irradiation. This formulation was highly stable and well tolerated both *in vivo* and *in vitro*, displaying cytotoxicity only after laser stimulation in a NP dose and laser intensity dependent fashion by causing hyperthermia. In *in vivo* experiment, aMG1-PEG-HNPs were able to accumulate in xenografted liver CRC metastasis, making them visible to MRI and making the tumours clearly detectable, and after laser irradiation, they were able to cause extensive necrosis of the tumor foci they accumulated in.

#### 2.4.8. Nanovectors for Multimodal Therapies against CRC

Multimodal therapies that combine physical and pharmacological therapeutic approaches offer the opportunity to achieve novel synergies in tumor treatment.

In particular, the recent FDA approval of cytotoxic antibodies expressed on regulatory lymphocytes (Tregs) such as anti-CTLA-4 ipilimumab and anti-PD-L1 atezolizumab, in the treatment of CRC inspired the use of these new tools in nanomedicine, combining treatments able to induce immunogenic cell death (ICD) and with their Treg suppressive action.

In particular, a recent study investigated the use of PEGylated upconversion nanoparticles (PEG-UCNPs) loaded with a NIR photosensitizer (Ce6) and a toll-like receptor 7 agonist (R837). This system was able to accumulate into *in vivo* implanted tumours and cause their PDT-mediated ablation upon NIR irradiation (980 nm). This in turn, caused the release of tumor-associated antigens (TAAgs). This in combination with R836 CTLA-4 checkpoint blockade by specific antibodies could induce a strong immune response. This proof of concept was confirmed by *in vitro* experiments on CT26 and dendritic cells (DCs) cocultures, in which DCs activation was highly enhanced after tumor cells treatment with UCNPs-Ce6-R837 and subsequent NIR irradiation. This was further reinforced by different *in vivo* experiments, achieving the complete primary tumor ablation after NPs treatment followed by PDT, which also caused reduction and removal of even distant tumours, increasing the percentage of CD8+ infiltrated cells. Furthermore, this treatment was able to induce antitumor memory as demonstrated by higher subject survival after tumor rechallenge [71].

In a similar study, Duan et al. employed phospholipid and zinc based self-assembling PEGylated nanoparticles to load oxaliplatin and dihydroartemisinin (DHA). Both these drugs are highly cytotoxic and act primarily by producing ROSs, which induce cells apoptosis. This system allowed good stabilization of the otherwise quickly inactivated DHA and showed good cytotoxicity on CRC cell lines, inducing apoptosis and the release of immunogenic death-associated molecular patterns (DAMPs) through immunogenic cell death (ICD). The release of these molecules was then hypothesized to be potential trigger for an antitumor immune response. Therefore, after demonstrating efficient tumor accumulation *in vivo*, this system demonstrated the capability of inducing pro-inflammatory M1 macrophage and CD8+ T-cell accumulation in immune-competent mice xenografted with CRC cells, with an increased production of pro-inflammatory cytokines such as IFN-*γ*. This effect was further potentiated by the double treatment of NPs and anti-PD-L1 antibody, and mice were able to develop antitumor antigen-specific immune memory, as demonstrated by the lack of tumor re-formation after rechallenge with the same cell line. On the contrary, when mice were implanted with different tumor cells, no immune response was observed [72].

In another recent study [73], EGFR-targeted cerasomes carrying the NIR label IRDye800CW, gadolinium chelated as the MRI imaging contrast agent, and porphyrin as the PDT sensitizer (EGFR-CPIG) were studied. This formulation allows for multimodal imaging through fluorescence and MRI. The use of these two complementary imaging modalities allows us to overcome the limitation of either one used singularly. Furthermore, the use of PDT is able to induce tumor ablation and potentially trigger an antitumor immune response. This immunogenic potential can potentially be enhanced by the use of checkpoint blockade therapeutic agents such as the recently clinically approved anti-PD-L1 antibodies. This system showed *in vitro* toxicity dependent on 650 nm laser irradiation time and intensity after NP administration on CRC cells. Furthermore, EGFR-CPIG NPs were able to accumulate in tumor in an ectopic CRC murine model. Furthermore, this platform allowed for tumor detection through MRI and FMI imaging compared to the untargeted formulation demonstrating how EGFR active targeting can substantially improve NPs tumor tropism. The use of PDT after EGFR-CPIG and anti-PD-L1 antibodies IV administration in a CRC murine model demonstrated a synergistic action of these two therapies combined, causing complete tumor eradication with no relapses over time. Tumor ablation was also observed with the untargeted CPIG formulation plus PDT and PD-L1, but relapses were still occurring over time, suggesting that the active targeting allows NPs to reach a critical tumor concentration able to achieve complete remission. Finally, this multimodal treatment in murine models did not cause any alteration in any of the major organs (lungs, heart, kidneys, liver, and brain), nor in the number of circulating RBCs, WBCs, and platelets, and there was no difference in the animal weight gain over time, making it a well systemically tolerated platform.

These systems are a remarkable example of how nanoparticles-mediated physical treatment can synergize with novel pharmacological therapies, obtaining effectiveness that is more than just the sum of the single treatments.

However, nanoparticles alone have also been demonstrated to be able to induce an antitumor immune reaction.

This latter approach was developed by Xu et al. [74]. This group formulated PEGylated mesoporous ruthenium nanoparticles loaded with the anticancer fluorescent peptide RBT and actively targeted with a bispecific antibody against CEA and CD16 (HMRu@RBT–SS–Fc). Ruthenium nanoparticles are excellent photosensitizers used in photothermal treatment since this material is able to produce ROS upon NIR light irradiation, as a source of ICD. The active targeting against CEA allows the particles to accumulate into the tumor site, while the anti-CD16 binding allows for activation of NK cells, priming the antitumor immune response. These nanoparticles demonstrated drug loading, slow drug release, concentration-dependent photothermal effect, ROSs production, and good stability during storage and after multiple NIR irradiation cycles. Furthermore, this formulation was endocytosed by 2D CRC cell lines proportionally to cells CEA expression, validating the NPs active targeting. HMRu@RBT-SS-Fc NPs were also more cytotoxic than free RBT and displayed good spheroid inhibition and penetration compared to the nontargeted formulation. Finally, these nanovectors were able to quickly accumulate in *in vivo* CRC models, exerting notable tumor-growth reduction and causing NK cells activation, especially after photothermal treatment. Of note, the induction of inflammation in the primary tumor was able to induce an immune reaction against an untreated secondary tumor that was not irradiated.

## 3. Conclusions and Future Perspectives

The field of nanotechnology is wide, highly heterogeneous, and quickly evolving. Hence, the necessity of this review to list, classify, and evaluate the latest trends of nanovectors development for CRC, as well as their preclinical and clinical testing by presenting examples of recent nanovectors formulations.

In the last years, the field of nanotechnology applied to CRC evolved significantly and was able to adapt and complement the latest advances in tumor diagnosis therapy that go far beyond the traditional paradigm of nanoparticles as simple drug delivery vectors, often combining it with completely new concepts and approaches in a synergistic way, as exemplified above.

NPs are still holding great promise in their applications to tumor detection and treatment, as testified by the great deal of work presented above. However, many nanoparticle formulations still suffer from some design limitations that are yet to be completely assessed. For example, many NP platforms show relatively low overall targeting efficiency, with most of the administered dose still retained from RES in the liver and spleen. The use of stealth-inducing polymers such as PEG and the use of active targeting moieties only demonstrated limited benefit in improving the NPs pharmacokinetics. Furthermore, many studies still do not provide quantification of global biodistribution, *de facto* ignoring this pressing issue. Therefore, the development of new design paradigms for NPs is sorely needed and could give great benefit to the development of innovative nanovectors.

In particular, the concept of biomimicry completely changed the potential of nanosystems applications [75, 76]. The use of not using only biological or biotechnological molecules, but of entire cellular components (and in particular of the plasma membranes and their receptors) as materials can make the nanosystems able to recapitulate the complexity of the biomaterials they are formulated from. This means that a nanosystem could, in theory, explicate the many different functions mediated by all the incorporated protein components, further enhancing the multitasking potential of the nanosystems. The use of these cell-like nanovectors also provides unprecedented opportunities to exploit intercellular communication to achieve more specific targeting and even drug-free therapeutic action; also, these systems can potentially act as “cell-decoys” in order to block direct intercellular interaction or prevent signalling mediated by soluble molecules (e.g., cytokines).

However, in order to obtain standardized formulations, *ad hoc* procedures for cell culture and protein extraction and purification and formulation need to be set up and validated, especially in the perspective of a scaling up.

Furthermore, the revolutionizing applications of immunotherapy to cancer are another strategic field that can greatly benefit from the implementation of nanotechnology. Many studies already reported how the use of nanoparticle adjuvant can enhance the efficacy of antitumor vaccines. Furthermore, the improved delivery of biological cargoes such as ONTs offers the possibility of effectively reprogram tumor-associated immune cells by gene therapy, either *ex vivo* or *in vivo*.

Despite the remarkable progresses in development of more complex and efficient nanovectors, in the large majority of studies, the biological testing of nanoparticles still relies on 2D cell cultures and ectopic murine models of CRC. These preclinical models are well known and validated, but they give only limited insight into the potential clinical efficacy of the formulations in study. Namely, 2D cell culture is characterized by a simple and unrealistic environment in which CRC cell lines are forced to grow only on a surface. This condition can alter the cells gene expression and polarization, inducing a phenotype different from the one found in the actual CRC tissue. Furthermore, there is no extracellular matrix, no vascularization, and no immune cells, all important features in the composition and architecture of solid tumours. 2D culture also overexposes tumor cells to nutrients and to the treatment, potentially giving skewed information in regards of the formulation efficacy.

The *in vivo* models employed still rely on the ectopic grafting of tumor cell into mice, mostly by subcutaneous or intraperitoneal injection for primary tumor. For the simulation of metastasis, tumor cells are injected intrasplenically or IV to cause CRC cells deposition in the liver and lungs, respectively. In some sporadic cases, CRC is induced chemically by ingestion of oncogenic chemicals (e.g., azoxymethane) (Tables 1–3).

Murine models are of course much more similar to the CRC found in the clinic, since the animal model provides a tissue for CRC to grow in, provides a more realistic architecture, and recapitulates most of the biological barriers the nanoparticles will encounter after administration and can give some indication regarding NPs toxicity. However, these techniques are not free from caveats. When human CRC are implanted, the use of nude mice is required to avoid quick immune clearance of the cells, thus depriving the model of the important immune components of tumor development. The use of murine CRC cells in immune-competent mice is an alternative, but no human components are present; thus, raising issues of translational viability. The use of humanized or genetically modified mice is perhaps the gold standard in this regard, but these strains are extremely expensive and generated *ad hoc.* Furthermore, since in most cases CRC cells are engrafted in an ectopic tissue, the microenvironment in which the cells grow in is dissimilar from the colorectal one [77].

Finally, in both *in vitro* and *in vivo* models, only a single CRC cell line is present, meaning that all the present cells are genetically identical among individuals and within each one. This is simplistic, given the known genetic heterogeneity of tumor populations in the clinic.

In this context, the development of more realistic preclinical models is sorely needed. For instance, the employment of patients-derived 3D tumor models [78] may offer a bridge to gap the oversimplified 2D cell cultures and the limitations of *in vivo* models. These new designs are not intended to substitute but to complement the currently available techniques [79].

This paper also gave some examples how the induction of immunogenic cell death by physical and/or pharmacological treatment through nanoparticles can prime the immune response needed for the success of new established immunomodulating anticancer treatments. Finally, exosomes have shown the ability to induce antitumor immune reactions by repolarizing tumor-associated macrophages. The formulation of biomimetic particles able to replicate the action of exosomes without their issues in reproducibility is a very elegant and fascinating prospective.

In conclusion, nanotechnology, and nanomedicine in particular, stands in a unique strategic position at the crossroads of many fields of research, ranging from biotechnology, passing through oncology therapy and diagnostics, to immunology. This *fil rouge* linking so many disciplines holds the promise of innovating and advancing all these lines of investigation towards new applications not only by combining them but also by offering new elegant solution to face the great health challenges of our time.

## Figures and Tables

**Table 1:** Summary discussion of all the advantages and caveats for each of the design features and solution summarily presented in Figure 1.

Size	

Very small particles are quickly cleared through renal filtration and in cone cases can penetrate the nuclei causing high toxicity. Very large nanoparticles are not suitable for IV injection: their aggregation can induce capillaries clogging and embolism. 20–200 nm nanoparticles are considered the range to achieve EPR-mediated NPs passive targeting. Smaller particles more easily penetrate tissues parenchyma and display better mucoadhesion. Larger particles injected locally are more efficiently retained in the tissue, because they diffuse less. Some NPs have size-dependent physiochemical proprieties (e.g., fluorescence for quantum dots, SPR for gold NPs, and superparamagnetism for SPIONs).	

Shape	

Spherical particles are the most widely diffused shape because they provide minimal surface-to-volume ratio, minimizing the energy state in self-assembling formulations. For nonspherical particles, the hydrodynamic diameter more closely resembles the diameter of the smallest dimension, thus influencing biodistribution. Nonspherical NPs interact more efficiently with cells and mucosae along their largest dimension. Some NPs have shape-dependent intrinsic physiochemical proprieties (e.g., SPR for gold nanoconstructs and optical proprieties for carbon NPs).	

Surface features	

Charge	Highly positive NPs strongly interact with negatively charged cell membranes and enhance uptake. However, they are not suitable for systemic administration because they also interact with proteins and cells in blood, becoming quickly opsonized and cleared by RES, or aggregation and potential embolism. Highly positive NPs interact strongly with the extracellular matrix when injected locally, and with mucosae. Highly negative NPs are similarly quickly cleared by RES.
Roughness	NPs with a rough surface have higher surface and interact more with proteins when administered IV.
Active targeting moieties	Include: small molecules (e.g., mannose, folic acid, and synthetic oligopeptides), proteins (antibodies and antibody fragments, toxins binding sites, and receptor ligands such as transferrin and albumin), aptamers, and adhesion/housing molecules for biomimetic platforms. Allow for better tissue targeting, complementing passive targeting strategies.
Stealth-inducing molecules	Include a wide range of neutrally charged, highly hydrophilic natural and synthetic polymers (e.g., PEG, PEI, polyglycerols, hydrophilic polyacrylates, chitosan, and dextran) and “self' molecules for personalized biomimetic formulations. Increase the plasmatic half-life of administered NPs by slowing opsonisation, allowing more accumulation in the target tissue.
Label/drug binding scaffolds	Provide binding moieties for both drugs, photosensitizers, and imaging molecules.

Material	

Carbon NPs	Have specific optical proprieties. High potential for systemic toxicity.
Liposomes	Are widely studied and can load both hydrophilic and lipophilic molecules, including proteins. Are well tolerated and some liposomal formulations are already FDA-approved.
Solid lipid NPs	Provide high loading for lipophilic drugs and can modulate their release. Biodegradable.
Dendrimers	Good size control and multiplexing capabilities.
Polymeric NPs	Wide range of materials and material blends to accommodate many different molecules and functions (e.g., pH dependence, and biodegradability).
Gold NPs	Chemically inert, provide X-ray and radiotherapy enhancement, tunable SPR, Sonodynamic and photodynamic therapies, and SERS.
Superparamagnetic iron oxide NPs	Provide high contrast for MRI imaging, magnetic field-guided tissue accumulation, and magnetic hyperthermia. Biodegradable and well tolerated.
Quantum dots	High fluorescence yield and photostability. Concerns about systemic toxicity.

**Table 2:** Summary of the all discussed nanoparticle formulations used for colorectal cancer detection and imaging, for each formulation size, zeta potential, targeting strategy, administration route, payload, *in vitro* and *in vivo* models they have been tested on, the relative results, and references.

Nanovector	Size (AVG)	Charge	Targeting strategy	Payload	Administration route	Model	Results	References
Anti-CEA MoAB-PAMAM-NIR664-doped silica NPs	70 nm	—	EPR Anti-CEA MoAB	NIR664	IV	*In vitro*: LS174 T, LoVo and HCT116 cell cultures *In vivo*: LS174 T subcutaneously injected in nude mice.	*In vitro*: Increased uptake compared with untargeted formulation. *In vivo*: Increased tumor-associated fluorescence compared to untargeted formulation.	[43]

FMSNs-UEA1	75 nm	–13 mV	EPR Α-L-fucose targeting	FITC	Topical	*In vitro*: HCT116 (*α*-L-fucose +) and Caco-2 (*α*-L-fucose +) cell lines *Ex vivo*: colonic mucus from A/J mice and colon tissue from DSS/AOM-induced CRC bearing A/J mice *In vivo*: DSS/AOM-induced CRC in A/J mice	*In vitro*: targeting of *α*-L-fucose + cell lines Ex vivo: good stability in mucus and binding to *α*-L-fucose + tissue. *In vivo*: marked NPs binding to CRC regions demonstrated by endomicroscopy	[44]

P(PE-PLLA)	103 nm	–30 mV	CEA active targeting	Fluorescent dye: ICG	IV (biodistribution) Intraluminal administration	*In vitro*: SW480 and HT29 cell lines and CAM-engrafted cells *In vivo*: BALB/c male mice (biodistribution, and LS174 t cells intracolinically implanted in nude BALB/c male mice	*In vitro*: CEA expression and anti-CEA AB (targeting moiety) dependent tumor binding *In vivo*: wide biodistribution after IV administration, good CRC detection after topical administration	[45]

HPMA-EPPT1-IR783	—	—	uMUC-1 active targeting	NIRF dye: IR-783	Intraluminal administration	*In vitro*: uMUC-1(++) HT29, uMUC-1 (+) LS174 t and uMUC-1 (–) SW480 cell lines *Ex vivo*: CRC and healthy tissue patient matched samples *In vivo*: LS174 t or HT29 administered in the colon wall of athymic female nude mice.	*In vitr*o: uMUC-1 dependent conjugate tumor-binding of targeted formulation *Ex vivo*: selective binding to CRC tissues of targeted formulation. *In vivo*: good tumor binding and detection.	[46]
EGFR/VEGF-F-SERSA/B	350 nm	—	EGFR/VEGF active targeting	Fluorescent dye: AF610 SERS dyes: RITC and FITC (EGFR and VEGF, respectively)	Topical	*In vitro*: HT-29 CRC cell line retroviral-transfected with luciferase DNA *In vivo*: transfected HT-29 cells injected in BALB/c nude mice in the colon wall.	*In vitro*: efficient cell labelling proportional to cell density and administered dose. *In vivo*: multimodal detection of small tumours in real time with high sensitivity and EGFR/VEGF profiling.	[48]

CNPs	—	—	LN tropism and retention	—	Endoscopic submucosal administration	152 rectal cancer patients	Enhanced LNs detection, quicker surgical removal of more LNs, leading to better nodal staging	[50]

FA-3WJ-pRNA NPs	—	—	EPR FR*α* active targeting	Alexa fluor-647	IV	*In vitro*: KN20 and HT 29 cell lines *In vivo*: nude mice model of CRC liver and lung metastasis	*In vitro*: FA-dependent cell binding and uptake *In vivo*: CRC metastasis targeting and avoidance of healthy parenchyma.	[51]

CNPs	150 nm	—	LN tropism and retention	—	Endoscopic submucosal administration	74 CRC T1 and T2 patients	Enhanced tracking if sentinel lymph node with near 100% accuracy in metastasis labelling.	[80]

Abbreviations: Avg: average; CRC: colorectal cancer; DOX: doxorubicin; EPR: enhanced permeability and retention effect; IV: intravenous; SC: subcutaneous; LN: lymph node; PDT: Photodynamic treatment; PTT: photothermal treatment.

**Table 3:** Summary of all discussed nanoparticle formulations used for colorectal cancer therapy, for each formulation, size, zeta potential, targeting strategy, administration route, payload, *in vitro* and *in vivo* models they have been tested on, the relative results, and references.

Nanovector	Size (AVG)	Charge	Targeting strategy	Payload	Administration route	Model	Results	References
*α*CEA-MoAB-PEG-PLGA NPs	200 nm	–10 mV	EPR Anti-CEA antibody	Paclitaxel	IV	*In vitro*: SW480 (CEA–) and Caco-2 (CEA+) cell cultures.	*In vitro*: CEA dependent uptake and cytotoxicity	[26]

Doxorubicin-loaded ONT-conjugated AuNPs	23 nm	—	EPR (tissue retention)	Doxorubicin	Intratumoural	In vitro: SW480 cell cultures *In vivo*: GFP-transfected SW480 subcutaneously inoculated in BALB/c female mice	*In vitro*: enhanced DOX cytotoxicity and delivery to the nucleus *In vivo*: slower tumor growth compared to control	[34]

AuNPs	15 nm	–21 mV	EPR	—	IV	*In vitro*: SW629 cell cultures *In vivo*: SW620 subcutaneously inoculated in female BLAB/c mice	*In vitro*: reduction in fibrosis-inducing factors secretion *In vivo*: decrease in solid stress and improved perfusion; improved cisplatin action	[36]

Cu-Ci	—	—	EPR	Radiotherapy enhancement PDT	—	*In vitro*: SW620 cell cultures	*In vitro*: nanoparticle dose and radiation dose dependence of cell death by autophagy and apoptosis.	[37]

CP-NIC NPs	74 nm (length) 13 nm (width)	—	EPR	Nicosamide	IV	*In vitro*: HCT116 cell line *In vivo*: HCT-116 subcutaneously injected in male nude mice	*In vitro*: HCT116 cell toxicity similar to free drug, Wnt inhibition *In vivo*: tumor growth delay and increase in survival	[52]

MSNs-DM1@PDA-PEG-APt	170 nm	–11 mV	EPR Aptamer against EpCAM	Maytansine	IV	*In vitro*: SW480 and NCM460 cell cultures. *In vivo*: SW480 subcutaneously injected in BALB/c mice	*In vitro*: EpCAM dependent cytostatic and apoptotic effect of targeted NPS *In vivo*: reduction of tumor growth rate	[53]

FA-HBcAg-PAA-DOX NPs	35 nm	—	EPR FR*α* targeting pH-dependent release	Doxorubicin	—	*In vitro*: HT-29, Caco-2 and CCD-112 cell lines	*In vitro*: FR and FR*α* dependent uptake and cytotoxicity, cytosolic drug delivery.	[54]
Dex-SA-DOX-CDDP	40 nm	—16 mV	EPR	Doxorubicin Cisplatin	IV	*In vitro*: CT26 cell lines *In vivo*: CT26 cells subcutaneously injected in male BALB/c mice; DMH intraperitoneal injection in BALB/c nude mice.	*In vitro*: dose-dependent cell toxicity *In vivo*: reduction of tumor growth, induction apoptosis, reduced hepatotoxicity and cardiotoxicity	[55]

RBC-coated PLGA nanoparticles	150 nm	—	EPR	Gambogic acid (GA)	IV	*In vitro*: SW480 *In vivo*: Ectopic SW480 bearing BLAB/c bearing mice	*In vitro*: biocompatible, 48h cytotoxicity similar to free drug *In vivo*: Reduction in tumor size and increased survival	[56]

anti-EGFR-iRGD-RBCms- PLGA NPs	153 nm	—	EPR Anti-integrin rec. and anti-EGFR targeting	Gambogic acid	IV	*In vitro*: Caco-2, HT-29 (EGFFR+) and SW-480 cell cultures. *In vivo*: Caco-2 cells subcutaneously injected in BALB/c mice. Reduced tumor growth and increased survival	*In vitro*: enhanced HT-29 spheroids penetration, similar GA cytotoxicity compared with the free drug *In vivo*: enhanced tumor targeting capability compared to nonfunctionalized NPs	[57]

Anti CD113 MoAB – pPEG-PCL/malPEG-PCL	167 nm	–28 mV	EPR Active targeting against CD113	SN-38	IV	*In vitro*: HT-29, SW620, HCT116 cell cultures. *In vivo*: HCT116 subcutaneously injected in BALB/c female mice	*In vitro*: selective toxicity on CD113 high cells only for actively targeted NPS *In vivo*: reduction in CD113 high tumor and histological reduction of CD113 + cells	[59]

PPDC nanoparticles	105 nm	—	EPR	Sorafenib CPT	IV	*In vitro*: HT-29 cell cultures (2D and spheroids) *In vivo*: HT-29 subcutaneously injected in BALB/c nude mice	*In vitro*: MMP-espression-dependent drug release and cytotoxic effect *In vivo*: Inhibition of tumor growth, reduced vascularization, and tumor necrosis	[62]
NKG2D-IL-21 dextran NPs	200–400 nm	40 mV	EPR	dsNKG2D-IL-21 plasmids	IV	*In vitro*: CT-26, NIH-3T3 and RAW264.7 cell lines *Ex vivo*: co-culture of above mentioned cell lines with spleen-derived mononuclear cells *In vivo*: CT-26 cells subcutaneously injected in BALB/c mice.	*In vitro* and *ex vivo*: efficient transfection, induction of immune cells activation. *In vivo*: Accumulation in tumor tissue, efficient cells transfection, and induction of immune response leading to tumor growth suppression.	[64]

RRHPC/PF33/pDNA	127 nm	–23 mV	Active targeting against CD44 and integrin *αvβ3*	TRAIL pDNA	Intraperitoneal	*In vitro*: SW480 tumor cell line *In vivo*: SW480 cell intraperitoneally injected in BALB/c female nude mice.	*In vitro*: Efficient targeting, transfection and apoptosis induction of tumor cell lines. *In vivo*: reduced tumor weight and reduced number of tumor nodules.	[65]

MSNs-anti-miR-155-PDA-AS1411	170 nm	–15 mN	EPR Anti-nuclein aptamer	Anti-miR 155 ONT	IV	*In vitro*: SW480, HT-29, SW-620, LoVo and Caco-2 cell lines. *In vitro*: BALB/c nude mice subcutaneously injected with SW480 cells	*In vitro*: aptamer-dependent uptake and anti-miR155 ONT -dependent toxicity, inhibition of colony formation *In vivo*: tumor targeting and growth inhibition; synergistic effect with 5-FU	[66]

miRNA-139-NPs	50–200 nm	—	EPR	miRNA-139	IV	*In vitro*: HCT-116 and LoVo cell lines. *In vivo*: HCT-116 subcutaneously injected in nude BALB/c mice; HCT-116 tumours implanted in nude mice colon	*In vitro*: miRNA-139 dependent toxicity *In vivo*: decrease in tumor growth and metastasis; increase in survival	[67]

SP-OA-CS	143 nm	–33 mV	EPR Mannose rec. targeting Hyaluronan rec. targeting	miR-20	IV	*In vitro*: LSECs isolated from Balb/c mice activated with tumor conditioned medium *In vivo*: C26 CRC cell administered by intra-splenic injection to produce CRC liver metastasis.	*In vitro*: efficient LSECs transfection, reduced expression of target proteins regulated by miR-20, reduced LSECs migration. *In vivo*:efficient LSEC targeting, reduced number of metastatic foci and reduced LSECs infiltration.	[68]

pRLN/pPD-L1 trap LCPs	186 nm	–6.8 mV	Active targeting against Sig-1R	pRLNpPD-L1 trap	IV	*In vitro*: CT26-F3 cell cultures. *In vivo*: CT26-F3 intrasplenically injected in male or female BALB/c and C57/BL6 mice	*In vitro*: efficient targeting and transfection. *In vivo*: Efficient targeting, transfection and modulation of the metastasis microenvironment towards a pro-inflammatory anticancer phenotype. Synergistic action with checkpoint inhibition. Decreased tumor growth and increased survival.	[69]

Gly@Cy7-Si-DOX NPs	80–120 nm	–30 mV to –80 mV	EPR	Cy7 DOX	IV	*In vitro*: bone marrow and HT29 cell cultures. *In vivo*: HT-29 cells subcutaneously injected in BALB/c athymic nude mice.	*In vitro*: NIR and pH dependent cytotoxicity. *In vivo*: tumor accumulation, NIR dependent therapeutic effect, tumor destruction, and improved survival	[70]

UCNPs-Ce6-R837	80 nm	–13 mV	EPR (tissue retention)	R837 Ce6 PDT-photosensitizers	Intratumoural	*In vitro*: CT26 cell lines alone on in trans-well co-culture with DCs. *In vivo*: CT26 subcutaneous implantation in BALB/c female mice	*In vitro*: NIR-dependent cell toxicity and DC activation *In vivo*: NIR-dependent primary tumor ablation, increased distal tumours reduction, and antitumor immune memory development	[71]
HMRu@RBT–SS–Fc	150 nm	+15 mV	EPR Active targeting against CEA and CD16	RBT	IV	*In vitro*: CT26, Caco-2, SW480, HCT116, CT-26 transfected with CEA cell lines and spheroids. *In vivo*: CT-26 cells transfected with CEA subcutaneously injected in BALB/c female mice	*In vitro*: Efficient active targeting and endocytosis by CEA expressing cells, uptake and photothermal dependent cytotoxicity *In vivo*: efficient tumor targeting and killing upon photothermal treatment; elicitation of immune reaction against secondary nonirradiated tumor.	[71]

Ox Pt-bp/chol-DHA	70–100 nm	–21 to –13 mV	EPR	OxPtDHA	Intraperitoneal	*In vitro*: CT26 and MC38 cell cultures *In vivo*: SD/CD rats, CT26 subcutaneously injected in BALB/c mice and MC38 subcutaneously injected in C57Bl/6 mice	*In vitro*: Dose-dependent cell death, induction of apoptosis and release of DAMPs. *In vivo*: RES avoidance, tumor growth inhibition (especially with PD-L1) blockade and acquisition of antigen-specific immune memory.	[72]

EGFR-CPIG	80–100 nm	40 mV	EPR Active targeting against EGFR	Porphyrin (PDT) IRDye800CW (MFI) DOTA-Gd (MRI)	IV	*In vitro*: CT26 tumor cell lines. *In vivo*: CT26 tumor cells subcutaneously injected in BALB/c male mice.	*In vitro*: laser intensity and irradiation time-dependent toxicity. *In vivo*: tumor growth suppression and eradication, immune cells activation after PDT treatment.	[73]

TCL-PDA NPs	245 nm	–24 mV	LNs tropism	TCL (providing tumor-associated antigens)	SC	*In vitro*: BMDCs cells isolated from C57BL/6 mice. *In vivo*: MC38 cells subcutaneously administerd in C57BL/6 mice	*In vitro*: BMDCs activation, antigen presentation, pro-inflammatory cytokine secretion. *In vivo*: Adaptive immune antitumor response, antitumor immune memory development. Substantial quenching of tumor growth.	[81]
aMG1-PEG-HNPs	50 nm ca.	—	EOR Active targeting against MG1	PTT MRI	IV	*In vitro*: CC-531 cell cultures *In vivo*: Surgical inoculation of tumours in the liver of Wistar rats	*In vitro*: MG1-dependent uptake, light-dependent cytotoxicity and increased MRI signal contrast. *In vivo*: tumor accumulation and detection through MRI, PTT tumor ablation upon 800 nm laser irradiation	[82]

Abbreviations: Avg: average; CRC: colorectal cancer; DOX: doxorubicin; EPR: enhanced permeability and retention effect; IV: intravenous; SC: subcutaneous; LN: lymph node; PDT: Photodynamic treatment; PTT: photothermal treatment.

## List of Abbreviations

5-FU: 5-Fluorouracil
AOM: Azoxymethane
APC: Antigen presenting cell
APts: Aptamers
AuNPs: Gold nanoparticles
BMDC: Bone marrow-derived dendritic cells
CAM: Embryo chorioallantoic membrane
CEA: Carcinoembryonic antigen
CNPs: Carbon nanoparticles
CRC: Colorectal cancer
CTLA-4: Cytotoxic T-lymphocyte Antigen-4
DAMPs: Death-associated molecular patterns
DC: Dendritic Cell
DHA: Dihydroartemisinin
DSS: Dextran sodium sulphate
EGFR: Epidermal growth factor receptor
EpCAM: Epithelial cell adhesion molecule
EPR: Enhanced permeability and retention
FDA: Food and Drug Administration
FITC: Fluorescein
FOLFIRI: 5-FU/leucovorin + irinotecan
FOLFIRINOX: 5-FU/leucovorin + oxaliplatin and irinotecan
FOLFOX: 5-FU/leucovorin + oxaliplatin
FR*α*: Folate receptor alpha
GA: Gambogic acid
gFOBT: Guaiac-based fecal occult blood test
GIT: Gastrointestinal tract
ICD: Immunogenic cell death
ICG: Indocyanine green
IFN*γ*: Interferon gamma
iFOBT: Immunochemical fecal occult blood test
IgG1: Immunoglobulin G1
IL: Interleukin
IV: Intravenous
LNs: Lymph nodes
mABs: Monoclonal antibodies
MDI: Maytansine
MDR: Multidrug resistance
MHC: Major histocompatibility complex
miRNA: Micro-RNA
MPS: Mononuclear phagocyte system
NIR: Near-infrared
NPs: Nanoparticles
ONT: Oligonucleotide
PAMAM: Polyamidoamine
PDA: Polydopamine
PD-L1: Programmed death-ligand 1
PDT: Photodynamic treatment
PEG: Polyethylene glycol
PLGA: Poly lactic-co-glycolic acid
PTT: Photothermal treatment
RBC: Red blood cell
RGD: Arginylglycylaspartic acid
ROS: Reactive oxygen species
SERS: Surface-enhanced Raman spectroscopy
SiRNA: Small interfering RNA
SLNs: Sentinel lymph nodes
SPIONs: Superparamagnetic iron oxide nanoparticles
SPR: Surface plasmonic resonanace
TAM: Tumor-associated macrophages
TCL: Tumor cell lysate
TGF-*β*: Tissue growth factor beta
TNBS: 2,4,6-Trinitrobenzenesulfonic Acid
TNF*α*: Tumor necrosis factor alpha
UEA1: Ulex europeaus agglutinin-1
uMUC-1: Under-glycosylated mucin-1 antigen
VEGFA: Vascular endothelial growth factor A
VEGFR: Vascular endothelial growth factor receptor.
